# Role of *Plasmodium berghei* ookinete surface and oocyst capsule protein, a novel oocyst capsule-associated protein, in ookinete motility

**DOI:** 10.1186/s13071-021-04868-2

**Published:** 2021-07-21

**Authors:** Kazuhiko Nakayama, Yuta Kimura, Yu Kitahara, Akira Soga, Asako Haraguchi, Jun Hakozaki, Makoto Sugiyama, Kodai Kusakisako, Shinya Fukumoto, Hiromi Ikadai

**Affiliations:** 1 Laboratory of Veterinary Parasitology, School of Veterinary Medicine, Kitasato University, Towada, Aomori 034-8628 Japan; 2 National Research Center for Protozoan Diseases, Obihiro University of Agriculture and Veterinary Medicine, Inada, Obihiro 080-8555 Japan; 3 Laboratory of Veterinary Anatomy, School of Veterinary Medicine, Kitasato University, Towada, Aomori 034-8628 Japan

**Keywords:** *Plasmodium berghei*, Ookinete, Oocyst, Knock out parasite, Transmission block

## Abstract

**Background:**

*Plasmodium* sp., which causes malaria, must first develop in mosquitoes before being transmitted. Upon ingesting infected blood, gametes form in the mosquito lumen, followed by fertilization and differentiation of the resulting zygotes into motile ookinetes. Within 24 h of blood ingestion, these ookinetes traverse mosquito epithelial cells and lodge below the midgut basal lamina, where they differentiate into sessile oocysts that are protected by a capsule.

**Methods:**

We identified an ookinete surface and oocyst capsule protein (OSCP) that is involved in ookinete motility as well as oocyst capsule formation.

**Results:**

We found that knockout of OSCP in parasite decreases ookinete gliding motility and gradually reduces the number of oocysts. On day 15 after blood ingestion, the oocyst wall was significantly thinner. Moreover, adding anti-OSCP antibodies decreased the gliding speed of wild-type ookinetes *in vitro*. Adding anti-OSCP antibodies to an infected blood meal also resulted in decreased oocyst formation.

**Conclusion:**

These findings may be useful for the development of a transmission-blocking tool for malaria.

**Graphical abstract:**

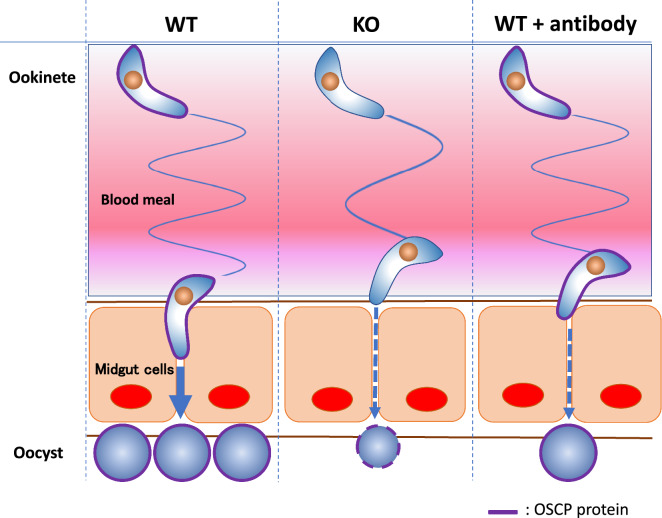

## Background

Malaria is among the world’s deadliest infectious diseases. In 2018, over 200 million people were infected and over 400,000 people died [1]. While significant advances have been made in the fight against malaria, progress has slowed in recent years mainly because of the prevalence of drug-resistant parasites and insecticide-resistant mosquitoes. To conquer malaria, it is vital to understand the biology of parasite transmission better and develop new tools for fighting the disease.

After the mosquito ingests an infected blood meal, parasites form gametes, followed by fertilization and differentiation of the resulting zygotes into motile ookinetes that traverse the midgut epithelium and lodge below the midgut basal lamina. It takes > 20 h from blood ingestion until the ookinete arrives at the midgut basal lamina. During this time, the parasites are attacked by both the mammalian and mosquito immune systems [2]. As a result, parasite numbers decrease by over a thousand fold, creating a potent bottleneck [3, 4]. The ookinete then differentiates into an oocyst and produces a capsule that helps it avoid the mosquito’s immune response. Within the capsule, many sporozoites are formed and released into the hemolymph, thus increasing parasite numbers by several thousand fold [5].

The mosquito mounts a strong immune response against the invading parasite [6, 7], and *Plasmodium sp.* produces a number of proteins to protect itself, some of which are expressed on the ookinete’s surface. The functions of P25/P28, P47, guanylate cyclase β (GCβ), putative secreted ookinete surface protein (PSOP25), *Plasmodium* invasion of mosquito midgut screen candidate 2 (PIMMS2), and PIMMS43 surface proteins have been discussed in existing literature [2, 8–10]. P25/P28, P47, and PIMMS43 are mainly expressed to protect the ookinete from the mosquito’s immune defense, while PIMMS2 is involved in protecting the ookinete while traversing the midgut cells. On the other hand, GCβ is mainly involved in the ookinete’s movement ability, and PSOP25 functions in ookinete maturation. Among the proteins that accumulate in the microneme of the ookinete, the functions of *Plasmodium* perforin-like protein 3–5 (PPLP3-5), secreted ookinete adhesive protein (SOAP), circumsporozoite- and TRAP-related protein (CTRP), cell-traversal protein for ookinetes and sporozoites (CelTOS), and chitinase have also been described [2, 13–22]. PPLP3-5, SOAP, and CelTOS aid in traversal of the midgut. CTRP is involved in the ookinete’s movement ability, while chitinase assists in traversal of the peritrophic matrix (PM), a chitin-containing layer surrounding the blood bolus.

The oocyst capsule is composed of mosquito-derived proteins, including laminin, matrix metalloprotease 1 (MMP1), and lysozyme c-1 (LYSC1) [23], and parasite-derived proteins such as *Plasmodium berghei* oocyst capsule protein 380 (PbCap380) and oocyst capsule-associated protein 93 of *Plasmodium berghei* (PbCap93) [23–25]. Knockout of the PbCap380 or PbCap93 genes results in decreased oocyst and sporozoite numbers [24, 25].

We focused on the transformation in the early steps of capsule formation as the ookinete differentiates into the oocyst. In this study, we investigated the functions of the high molecular weight (494 kDa) ookinete surface and oocyst capsule protein (OSCP: PBANKA_1025100) from PlasmoDB (https://plasmodb.org/plasmo/app/). The OSCP gene was selected based on its expression on ookinete surface and oocyst capsule. Therefore, OSCP was expected to play a critical role in ookinete and oocyst stages. In this study, we characterized the role of OSCP in the ookinete stage and oocyst stage. Finally, we present a new candidate vaccine antigen with transmission-blocking properties.

## Methods

### Parasites, mice, and mosquitoes

For *P. berghei* infections, 6- to 8-week-old male BALB/c mice (SLC, Japan) were infected with either wild-type (WT) *P. berghei* (ANKA strain) or P. berghei (ANKA strain), which constitutively expresses GFP [26].

Anopheles stephensi (STE2 strain) mosquitoes were maintained at 27 °C and 80% relative humidity with a 14/10 h light/dark cycle in an insectary and fed 10% (w/v) sucrose solution.

For the mosquito infection experiments, *P. berghei*-infected mice having ten exflagellations per 10^4^ RBCs or more were used. In the mosquito-to-mouse infection experiment, we used mosquitoes infected with WT and knockout (KO) parasites (25 days after the mosquitoes had ingested the infected blood meal).

### Construction of OSCP-KO parasites

Procedures were as previously reported [24, 27], using PCR amplification with primers OSCP-F1 (5ʹ-CAA AAC AAT GTG GAT TTG TG-3ʹ) and OSCP-R1 (5ʹ-GGT TTT CAT TTT CCC TAA AAT C-3ʹ) and *P. berghei* genomic DNA as a template. The gene was cloned into the pCR-BluntII-TOPO vector (Thermo Fisher Scientific), resulting in the plasmid pOSCP. Subsequently, pOSCP was digested using *Acc* I. The digested pOSCP was inserted into the *hdhfr* expression cassette [28]. pOSCP (10 μg) was linearized with *Xho* I and electroporated into cultured *P. berghei* schizonts using Nucleofector II (Lonza, Basel, Switzerland). Transfected parasites were intravenously injected into male BALB/c mice that were then treated with pyrimethamine (70 μg/ml) 24 h later via drinking water.

PCRs with the following primer combinations were performed to detect the presence of recombinant parasites. T1: OSCP-F2 (5ʹ-CCA TAC CTT CAA GAT TAG ATG AC-3ʹ) with hDHFR-shDR (5ʹ-CTG TTA TAA TTA TGT TGT CTC TTC-3ʹ), T2: hDHFR-shDF (5ʹ-CGA AAA GAA TTA AGC TTA ACT C-3ʹ) with OSCP-R3 (5ʹ-GCA GAT CCG TCC GTT TAA C-3ʹ), and T3: OSCP-F2 with OSCP-R2.

To analyze OSCP expression during oocyst stage, using the Trizol reagent (Thermo Fisher Scientific, Waltham, MA, USA), total RNA was isolated from the mosquito at 10 days after infection. cDNA was synthesized using the ReverTra Ace kit (Toyobo, Osaka, Japan). PCR was performed using following primers OSCP-F3 (5ʹ-TCG AGA TGG ATG CAA AGA CTA GCA G-3ʹ) and OSCP-R3 (5ʹ-GAT TCA CTG AAG GCT CAG GTT TAC C-3ʹ).

### Anti-OSCP antibody preparation and purification

**Fig. 1:**
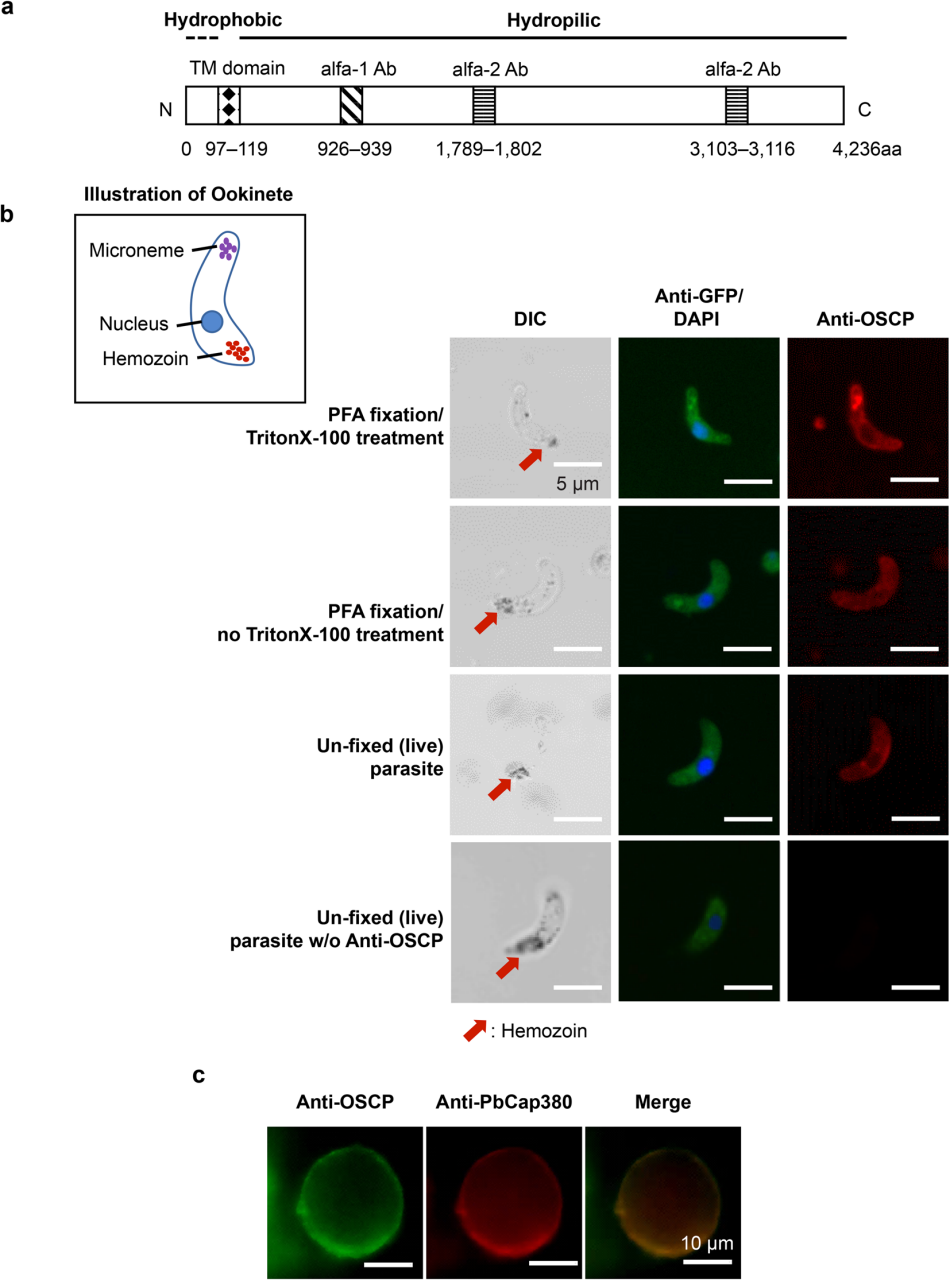
OSCP is expressed in the ookinete and oocyst stages. **a** Schematic representation of the OSCP amino acid sequence. Polyclonal antibodies were prepared against peptide-comprising amino acids 926 to 939 (anti-OSCP α-1); a mixture of peptides and amino acids 1,789–1,802 plus 3,103–3,116 (anti-OSCP α-2) antibodies is shown. **b** Immunofluorescence analysis of OSCP expression by ookinetes. OSCP localized to the ookinete surface. The ookinete parasite was labeled twice with antibodies against GFP (green: center panel) and OSCP (red: right panel), stained with the DAPI nuclear stain (blue), and showing differential interference contrast (DIC: left panel), as indicated. Negative control was performed as only anti-GFP antibody incubation on unfixed (live) parasites (w/o Anti-OSCP). *Scale-bars*: 5 µm. **c** OSCP is localized to the oocyst capsule. Images of an oocyst 15 days after the blood meal

Rabbit polyclonal antibodies were raised against the following OSCP protein domains: α1 (amino acids 926–939) and α2 (mixed amino acids 1789–1802 and 3103–3116) (Eurofins Genomics Inc., Tokyo, Japan) (Fig. 1a). We utilized the epitope selection service offered by Eurofins Genomics Inc. (Tokyo, Japan) for epitope selection. The OSCP amino acid sequence was divided into three segments on the basis of length. Among these, α1 domain in the first segment had the highest antigenicity. For the other two segments, antibodies against the highly antigenic regions of each were mixed to form α2. Antibodies were purified by saturated ammonium sulfate solution and Ab-Rapid SPiN EX (ProteNova, Japan). Ab-Rapid SPiN EX was performed following the manual until equilibration occurred. The purified antibodies were designated as anti-OSCP α1 and α2 antibodies. As a control, we also prepared non-immune rabbit antibodies by processing non-immune rabbit blood serum in the same manner.

## Blood-stage parasite and exflagellation analysis

Parasitemia and gametocyte sex ratio were determined by preparing blood smear specimens from infected mice, fixing these with 100% methanol and then performing Giemsa staining. Exflagellation of male gametocytes was quantified as previously described [23, 29]. Briefly, 2 μl of gametocyte-infected blood was obtained from the tail vein and mixed immediately with 38 μl of the complete ookinete culture medium (OKM: RPMI 1640 medium (Gibco, USA) with 20% heat-inactivated fetal bovine serum (Sigma-Aldrich, USA). The mixture was placed under a coverslip at room temperature (RT), and 5 min later, exflagellation centers were counted over the next 10 min using a phase contrast microscope. The number of exflagellation centers/10^4^ RBCs was measured per 8 μl using a Thomas hemocytometer (Nippon Rinsho Kikai Kogyo Co., Ltd., Japan).

### Ookinete culture and purification

Blood from mice with exflagellation of 10/10^4^ RBCs was obtained by heart puncture. Ten volumes of OKM was added to the blood and cultured for 24 h at 19 °C. The size and number of ookinetes was measured by taking a smear from the culture solution. Ookinetes were purified using a MidiMACS separator system (Miltenyi Biotec, Germany) as previously described [30]. A total of 3 ml culture was passed through three times before removing the column from the magnet. The ookinetes were recovered by passing 5 ml of OKM through the column. The purified ookinetes were centrifuged at 1,000 × * g* 10 min at 4 °C and then washed three times with PBS.

### Immunofluorescent assays (IFAs)

Cultured ookinetes were placed for 30 min at 4 °C on a MAS-coated adhesive slide glass (Matsunami, Japan), fixed with 4% paraformaldehyde (PFA)/PBS at RT for 10 min, and then washed three times with PBS for 5 min each time. After incubating for 10 min with 0.1 M glycine (Wako, Japan), they were washed three times with PBS for 5 min each time. Ookinetes were permeabilized for 10 min with 0.2% TritonX-100/PBS and washed with PBS for 10 min. No TritonX-100 was added when observing OSCP surface localization. Blocking was performed for 1 h at RT using 1% BSA/PBS. The primary antibody, anti-OSCP serum (α1 to α2 ratio 1:1) diluted 800-fold with PBS, was incubated for 1 h at RT and then washed three times with PBS for 5 min each. Non-immunized serum was used as negative control. Incubation with the secondary antibody, Alexa Fluor® 488 goat anti-rabbit IgG (H + L) (Thermo Fisher) diluted 1000-fold with PBS, was for 1 h at RT followed by three washes with PBS for 5 min each time. The parasites were mounted in SlowFade®Diamond Antifade Mountant with DAPI (Molecular probes, USA) and observed using an Eclipse E600 (Nikon, Japan) fluorescence microscope. The oocysts were observed by dissecting mosquitoes on day 15 after ingesting an infected blood meal, and staining was performed. Methanol fixation was performed, and after air-drying, the oocysts were treated with Image-iT FX Signal Enhancer solution (Thermo Fisher) for 30 min at 37 °C. After PBS washing, 400-fold-diluted anti-OSCP serum was added as the primary antibody and allowed to react for 2 h at 4 °C. After three PBS washes, Alexa Fluor® 488 goat anti-rabbit IgG (H + L) secondary antibody (Thermo Fisher) was added and allowed to react for 1 h at 4 °C. After three PBS washes, 1000-fold-diluted anti-PbCap380 rabbit serum was added as the primary antibody and allowed to react for 2 h at 4 °C. As the secondary antibody, Alexa Fluor® 568 goat anti-rabbit IgG (H + L) (Thermo Fisher) was added and allowed to react for 1 h at 4 °C. After three PBS washes and mounting, observation was performed with an Eclipse E600 (Nikon) fluorescence microscope.

### Ookinete locomotion

Ookinete movement was investigated by mixing ookinetes with Matrigel (Corning, USA) and then measuring displacement speed [10, 31]. Purified ookinete suspension and Matrigel were mixed in equal amounts, and then 15 µl drops were applied to glass slides. Glass covers (18 × 24 mm) were placed on top, and the specimens were left to stand for 20 min on a ThermoPlate (NHP-45 N; Nissin, Japan) at 19 °C. Next, a digital microscope (KH-8700; Hirox, Japan) was used to observe ookinete movement for 2 min. When imaging 10-min movement trajectories, WT and KO parasites were incubated for 30 min at 4 °C with MitoBright LT Red (Dojindo, Japan) diluted 1000-fold with PBS. Centrifugation was performed at 1000×*g* for 10 min at 4 °C and then washed with PBS. This process was performed twice. Then, as stated above, the specimens were mixed with Matrigel and left to stand. They were then observed and imaged with IX83 CellSens (Olympus, Japan). In the antibody reaction experiment, antibodies diluted with PBS were added to the purified ookinete suspension and mixed with an identical amount of Matrigel before undergoing the same process. Non-immune rabbit antibodies were used as the negative control.

### Oocyst number and size

Midgut oocyst numbers were counted using a Leica M205 FA (Leica, Germany) after staining the infected midguts with 0.5% mercurochrome/distilled water for 5 min and then washing with PBS for 5 min [32]. The midguts were also imaged with Leica Application Suite X (Leica, Germany). We randomly selected 60 oocysts from the imaged midguts and used Image J to measure oocyst size.

The ratio of normal to deformed oocysts was observed in mosquitoes on days 6 and 7 after the blood meal. Oocysts with an internal vacuole were considered deformed.

### Transmission-blocking (TB) experiment using antibodies

BALB/c mice were infected with GFP parasites by intraperitoneal injection of 10^6^ parasites. Mice that exhibited exflagellation of 10/10^4^ RBCs or more 5 days after infection were used for the experiments. In total, 50 female mosquitoes (Pre-group) were allowed to feed on the infected mouse for 15 min. This was followed by intravenous injection of 200 µg of anti-OSCP antibodies (α1:α2 = 1:1). After 5 min, another 50 mosquitoes (Post group) were allowed to feed for 15 min on the same mouse. Oocysts were counted 2 or 10 days later. For the TB experiment assayed 2 days after the blood meal, oocyst numbers were counted again after washing the guts with PBS for 1 min to remove any attached ookinetes.

### Transmission electron microscopy

On days 17 after the infected blood meal, mosquito midguts were fixed in 2.5% glutaraldehyde and 2% paraformaldehyde in 0.05 M sodium cacodylate buffer (pH 7.4) at 4 °C for 2 h. After rinsing, samples were post-fixed at RT in buffered 1% osmium tetroxide for 2 h and then dehydrated in ethanol and propylene oxide series and embedded in epoxy resin. Ultrathin sections were cut using an Ultracut N (Reichert-Nissei, Tokyo, Japan) and stained using uranyl acetate followed by lead citrate. Sections were examined using an H-7650 transmission electron microscope (Hitachi Ltd., Tokyo, Japan).

### Statistical analysis

Welch’s t-test was used to perform comparisons between groups (parasitemia, gametocytemia, number of ookinete, ookinete speed, oocyst size). The Mann-Whitney U test was used to compare the number of oocysts/midgut. All analyses were performed with GraphPad Prism software (GraphPad, San Diego, CA, USA).

## Results

### OSCP is expressed in ookinetes and is a component of the oocyst capsule

A PlasmoDB search performed for proteins expressed on the *P. berghei* ookinete surface and that are also part of the oocyst capsule led to the identification of OSCP (PBANKA_1025100). OSCP is predicted to have 4236 amino acids and has a molecular weight of 494 kDa. It has a predicted transmembrane domain in amino acids 97–119 (Fig. 1a). OSCP has orthologs with PF3D7_1417600 (amino acid homology: 60.8%) of *P. falciparum*, PKNH_1340400 (amino acid homology: 30.8%) of *P. knowlesi,* and PVP01_1331300 (amino acid homology: 33.1%) of *P. vivax.*

To investigate the localization of OSCP expression, we labeled ookinetes and oocysts with anti-OSCP antibodies using immunofluorescence assays (IFAs). These IFAs indicate whether OSCP is present in ookinetes.

The IFA images comparing ookinetes fixed with PFA and TritonX-100 treatment, fixed with PFA and no TritonX-100 treatment, and unfixed (live) parasites showed that OSCP was expressed on the ookinete surface (Fig. 1b). PbCap380 is a surface protein of the oocyst capsule [24], and the IFAs of day 15 oocysts using anti-OSCP and anti-PbCap380 antibodies indicated that OSCP co-localizes with PbCap380 (Fig. 1c).

### OSCP is not required for asexual growth

**Fig. 2:**
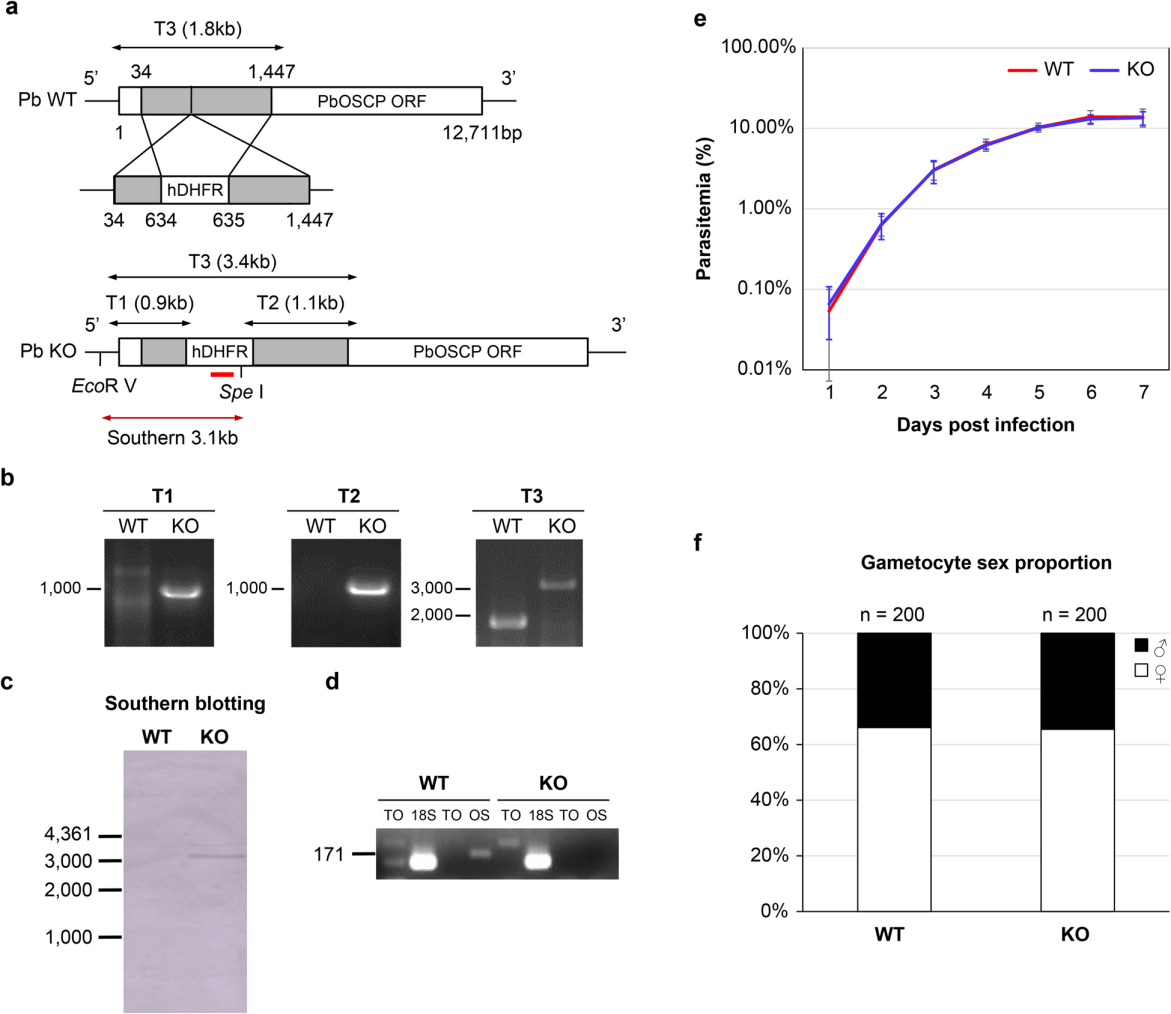
OSCP is not required for asexual growth.** a** Schematic illustration of OSCP gene knockout (KO) generation. A vector containing the hDHFR selection gene was used to disrupt the OSCP gene. Recombinant parasites were selected based on pyrimethamine resistance. Primers used for PCR analysis in (**b**) and the probe (red line) used for Southern blotting in [**c**] are indicated. **b** PCR analysis of WT and KO parasites. Primers are those shown in (**a**). **c** Southern blotting of WT and KO DNA cut with *Eco* R/*Spe* l and hybridized with the probe shown in [a] (red line). **d** Semi-quantified abundance of OSCP mRNA. Semi-quantitative RT-PCR was conducted using cDNA prepared from midguts of mosquitoes infected with either WT or KO parasites at 10 days post-infection. TO: total RNA; 18S: 18S RNA(133 bp); OS: OSCP (171 bp). (**e**) Comparison of WT and KO parasite growth in mice. Mice were infected by intravenous injection of 10^6^ parasites. Number of mice analyzed: *n* = 3 (WT) and *n* = 3 (KO). **f** Gametocyte sex ratio in mice infected with WT and KO parasites

As illustrated in Fig. 2a, we obtained KO parasites that were confirmed by PCR (Fig. 2b) and Southern blotting (Fig. 2c). Semi- quantitative RT-PCR verified that mRNA was not transcribed (Fig. 2d). The growth of WT and KO parasites in mice was indistinguishable (Fig. 2e), as was the gametocyte sex ratio (Fig. 2f). These results suggested that OSCP does not play an important role in the blood-stage development of the parasite.

### OSCP is involved in ookinete locomotion

**Fig. 3:**
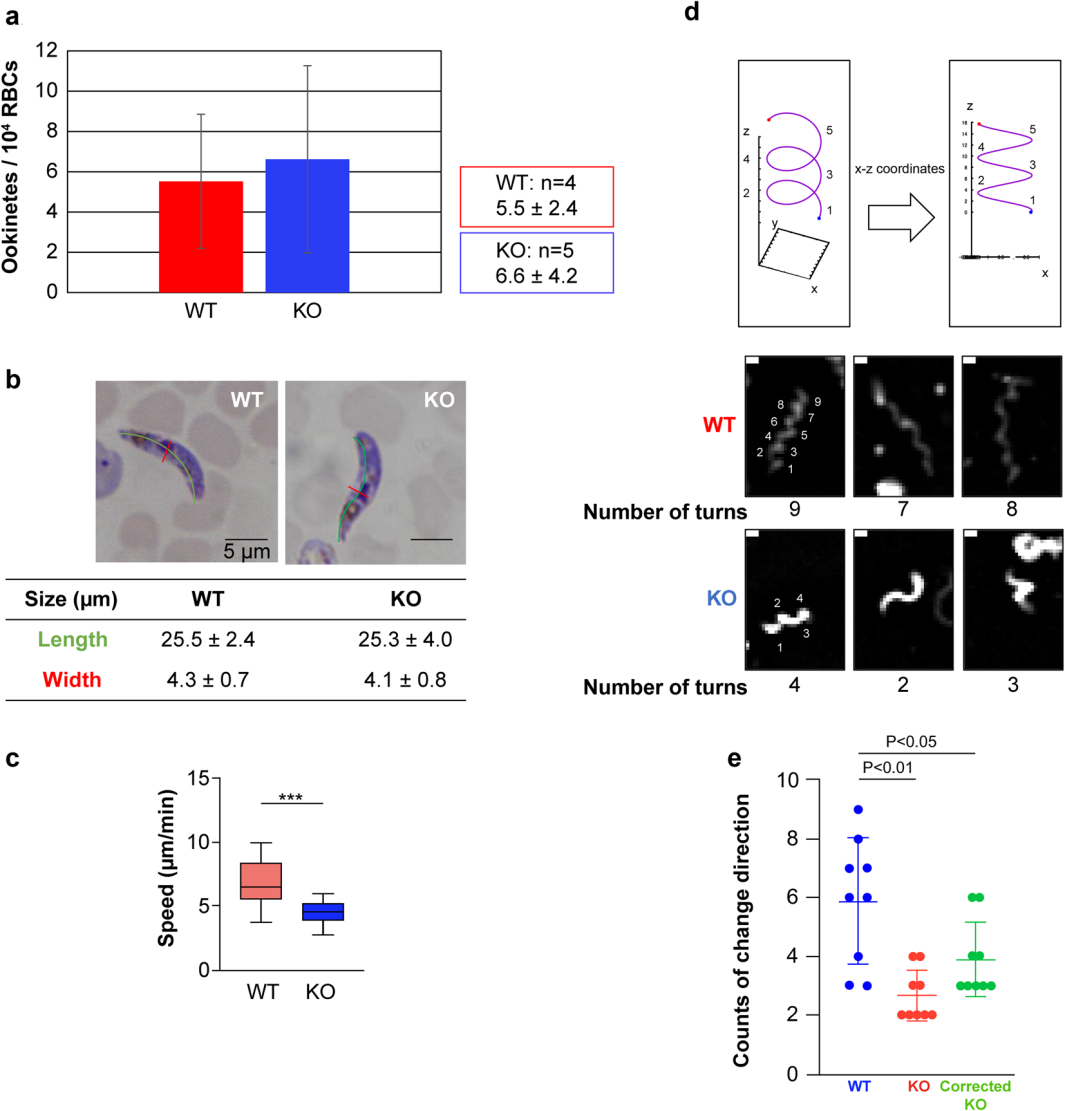
OSCP is involved in ookinete locomotion. **a** WT and KO ookinete formation *in vitro*. **b** Comparison of ookinete dimensions. Green line: length, red line: width. Number of ookinete analyzed: *n* = 20 (WT) and *n* = 20 (KO). **c** Comparison of ookinete gliding speed. Data pooled from 20 measurements for each WT and KO parasites. Welch’s t-test: ***P < 0.001 **d** Upper panels: Schematic diagram of spiral movement. Each turn around a spiral axis is comprised of two directional changes. Lower panels: examples of trajectories over 10 min. **e** Comparison of WT and KO ookinete number of turns. Corrected KO is WT ookinete speed/KO ookinete speed (6.74/4.53 = 1.49; panel **c**) multiplied by number of KO ookinete turns. The number of KO ookinete turns is significantly lower regardless of correction. Welch’s t-test: *P < 0.05 **P < 0.01.

We then investigated OSCP function in mosquito stages. There was no difference between the number of WT and KO ookinetes formed (Fig. 3a), and no differences were noted in ookinete size (Fig. 3b), indicating that OSCP is not involved in ookinete development or differentiation. Comparison of ookinete gliding on glass slides revealed that KO ookinete motility is significantly lower than WT ookinetes (Fig. 3c). Observations of ookinete trajectories over 10 min revealed that the number of turns performed by the KO ookinetes is significantly lower than that of WT ookinetes (Fig. 3d, e). This holds true even after adjusting for the lower KO ookinete locomotion speed (Fig. 3e).

### OSCP is involved in oocyst capsule formation and maintenance

**Fig. 4:**
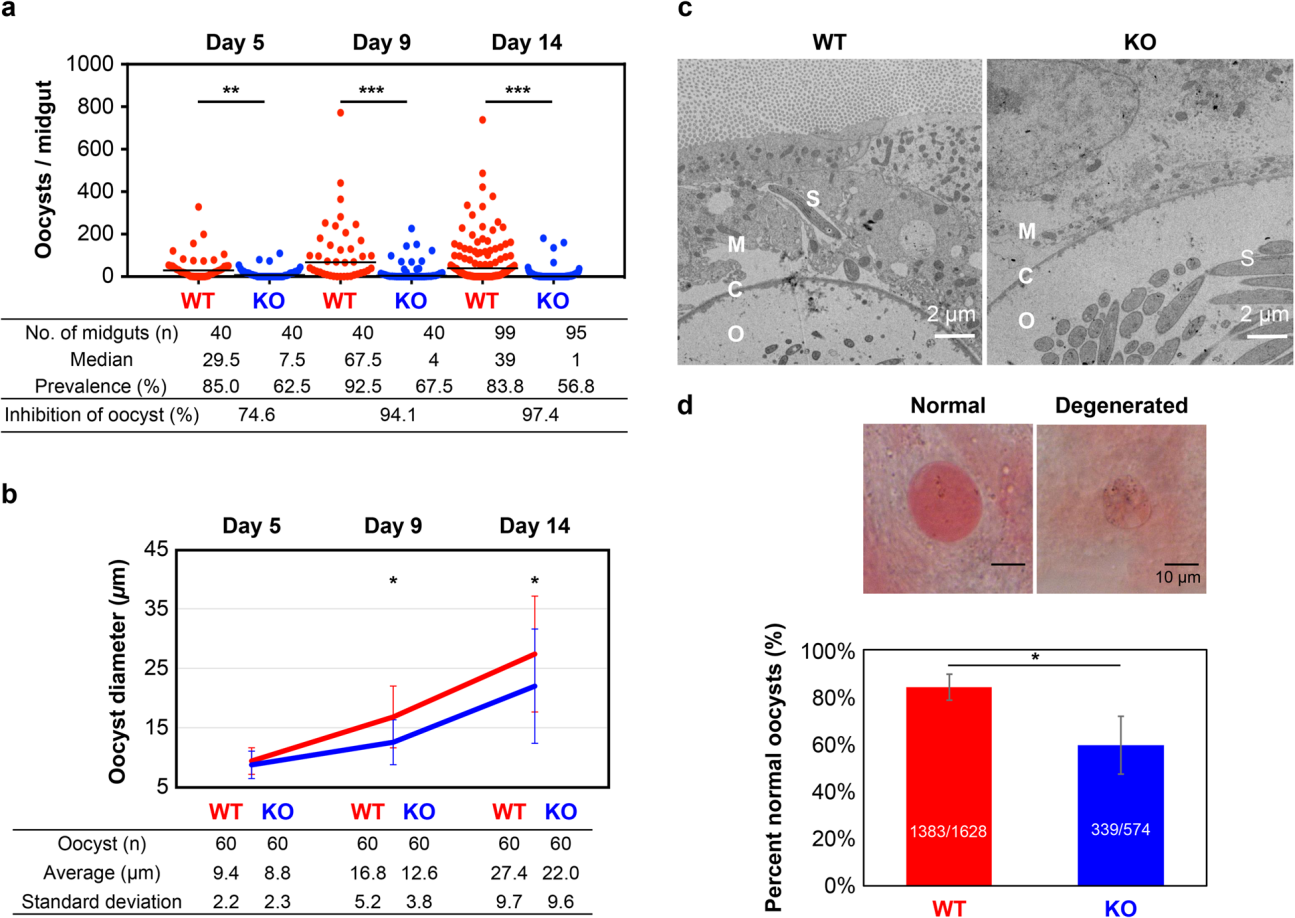
OSCP is involved in oocyst wall formation and maintenance.** a** Number of oocysts per midgut at 5, 9, and 14 days after an infected blood meal. The horizontal bars show the median value. The inhibition of oocyst number was calculated using (1-KO-oocyst median number/WT-oocyst median number) × 100. Number of independent experiments: day 5 and day 9 in duplicate and day 14 in triplicate. Mann–Whitney U test: **P < 0.01, ***P < 0.001. **b** Oocyst diameter per midgut at days 5, 9, and 14. Welch’s t-test: *P < 0.01. **c** Transmission electron microscopic images of oocysts at day 17. O: oocyst; C: oocyst capsule; M: mosquito tissue; S: sporozoite. **d** Upper panels (mercurochrome staining): examples of normal and degenerated oocysts. Lower panel: Proportion of WT and KO normal oocysts at day **6–7**. Mann-Whitney U test: *P < 0.05.

We compared WT and KO parasite infection rates and the number of oocysts formed at 5, 9, and 14 days after ingestion of an infectious blood meal. The number of oocysts formed was significantly reduced in KO infections compared to WT infections at the three time points measured (Fig. 4a). Moreover, on days 9 and 14, KO-oocyst diameter was significantly smaller than of WT oocysts (Fig. 4b). Transmission electron microscopy revealed that on day 17, KO oocysts exhibited wall thinning and indistinguishable internal structures. Sporozoites in KO oocysts differed in size and cell construction compared to sporozoites in WT oocysts. While some sporozoites of WT were observed outside of oocyst, sporozoites of KO were not fully matured in oocysts on day 17 (Fig. 4c). Furthermore, mercurochrome staining also showed a smaller proportion of normal oocysts (Fig. 4d). These results suggest that OSCP is involved in oocyst wall formation and maintenance. When mosquitoes were allowed to feed on naive mice 25 days after an infectious blood meal, they transmitted both WT and KO parasites, suggesting that OSCP is not essential for sporozoite function (data not shown).

### Anti-OSCP antibodies inhibit transmission to mosquitoes

**Fig. 5:**
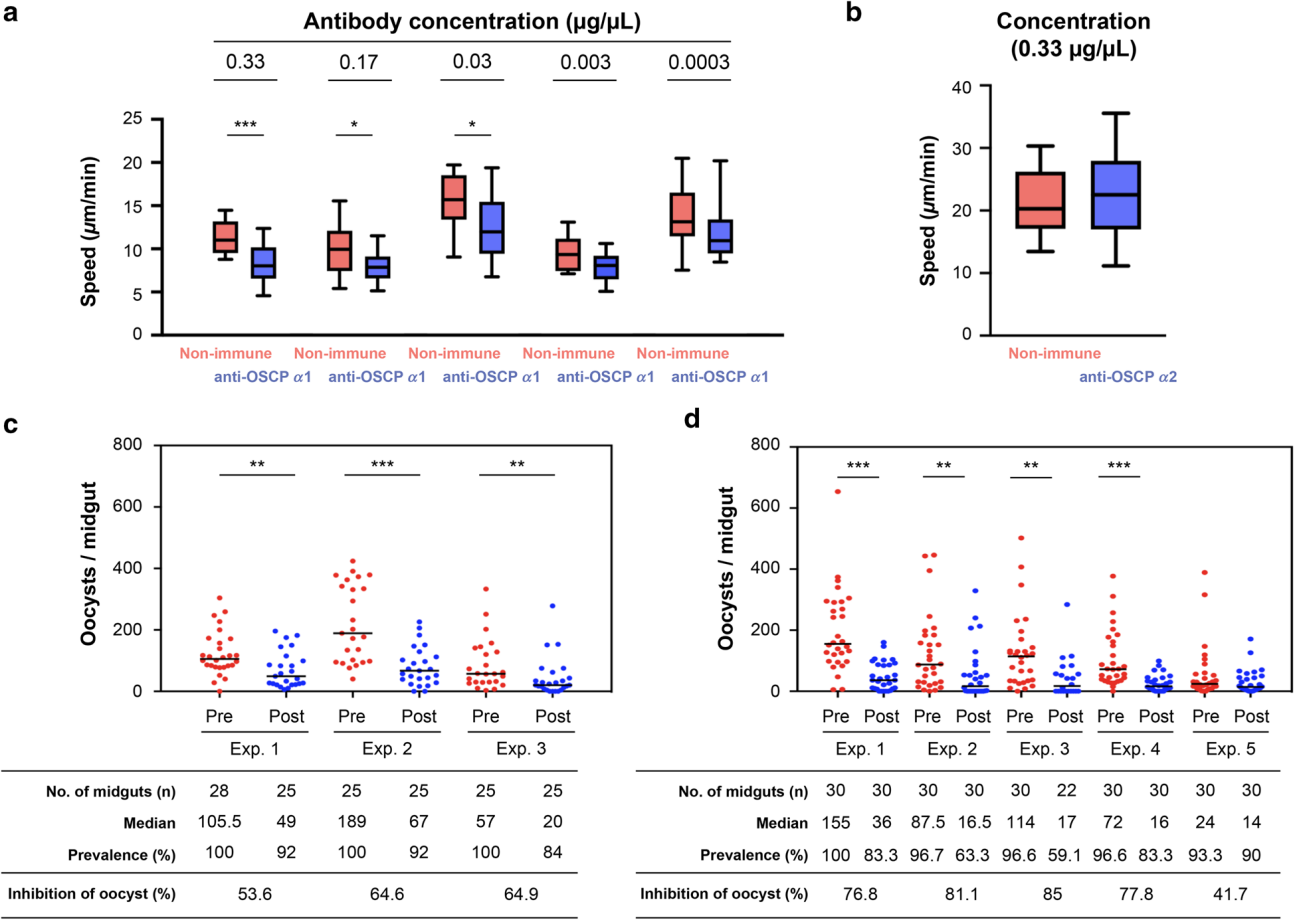
Anti-OSCP antibodies inhibit mosquito infection. **a** The anti-OSCP α1 antibody reduces ookinete gliding speed. Welch’s t-test: *P < 0.05, ***P < 0.001. **b** The anti-OSCP α2 antibody does not affect ookinete gliding speed. **c** and **d** Mosquitoes were fed on infected mice prior to (Pre) and then after (Post) intravenous injection of a mixture of equal amounts of α1 and α2 antibodies. Oocysts were counted at 2 (**c**) and 14 (**c**) days after feeding. Mann-Whitney U test: ***P < 0.001. Number of independent experiments in triplicate (**c**) and five times (**d**)

We investigated whether anti-OSCP-α1 and -α2 against epitopes presumed to be exposed to the parasite surface (Fig. 1a) can inhibit parasite function and transmission. Addition of the α1 antibody significantly decreased ookinete speed (Fig. 5a), while the addition of the α2 antibody did not affect speed (Fig. 5b).

To investigate whether a decrease in the speed of ookinete affects oocyst formation in mosquitoes, we passively immunized mice by intravenously injecting them with an equal mixture of α1 and α2 anti-OSCP antibodies. Antibody injection into mice imitates the immune response against OSCP antigens. Mosquitoes that fed on the blood of the same mouse prior to antibody injection served as controls. The antibodies showed an inhibitory effect on oocyst formation on day 2 after blood feeding (Fig. 5c), indicating that the antibodies act at the stage of ookinete entry into midgut cells. The experiments in Fig. 5d were performed to support the data (Fig. 5c) associated with the decrease in the number of oocysts due to the decrease in the speed of ookinete movement.

## Discussion

In this study, we investigated OSCP, a high molecular weight protein expressed in both the ookinete membrane and oocyst capsule. IFAs demonstrated that OSCP is expressed on the ookinete surface and also inside ookinetes after permeabilization. In OSCP-KO parasites, although no major differences were observed in ookinete growth and formation, movement speed decreased and the number of oocysts that formed was markedly lower. Thus, knocking out OSCP affected ookinete gliding. The PM represents a physical barrier between the fed blood and the midgut epithelia found in mosquito. The PM is an extracellular mesh composed of chitin, sugars, and protein [33]. When ookinetes pass through the blood as it is being ingested, because of the PM in the midgut space and midgut cells, they move forward more efficiently in a spiral trajectory instead of a straight-line trajectory, as in the case of an arrow [33]. Therefore, ookinetes move in a spiral manner from the midgut space to below the basal lamina of midgut cell. A previous *in vivo* experiment of mosquito midgut tissue and a previous *in vitro* experiment using Matrigel confirm these findings [33–35]. Ookinetes that move in a linear fashion are likely to lose significant amounts of energy and become trapped in the midgut tissue before reaching the area below the basal lamina from the midgut lumen. Ookinetes damage the midgut cells while traveling to the basal lamina, which activates the mosquito’s immune defenses, causing the ookinete to be eliminated [35]. Accordingly, ookinetes that move in a linear fashion exhibit decreased ability in infecting the mosquito. In fact, one study found that knocking out the IMC1h present below the ookinete cell membrane caused the ookinetes to move in a linear fashion, and the number of oocysts decreased [31]. In this case, because of the reduction in the speed of ookinete movement and the change in morphology, although it is unlikely that the linear motion directly caused the decrease in the number of oocysts, the change in the trajectory of motion could be one of the factors. In our study, the OSCP-KO parasite exhibited decreased movement speed as well as deviation in the normal spiral movement. The decreased number of turns in three-dimensional space noted in the KO parasite could have been due to the ookinete movement changing from a spiral to a linear pattern. In an *in vivo* experiment using mosquitoes, ookinetes that perform spiral movements were found to be involved in invasion below the midgut basal lamina [35]. Our ookinetes performed spiral movements in a three-dimensional space on the Matrigel-coated slide glass. For every two turns, the ookinetes turned once on a spiral-based axis (Fig. 3d). This indicates that OSCP may also be involved in the performance of spiral movements upon invasion of the midgut *in vivo*. These results therefore suggest that OSCP is involved in ookinete locomotion. Since no changes to ookinete morphology were observed in the OSCP-KO parasite, we inferred that this was because the altered movement trajectories were not due to abnormal adhesion to the extracellular matrix, which is required during ookinete movement. The results of a previous *in vivo* experiment also observed adhesion to the midgut cells was alongside rotational movement; a similar movement was observed in our experiment’s response [34, 35]. Moreover, our evaluation focused on spiral movement in order to investigate locomotion to the area under the midgut basal lamina.

Unlike CTRP, however, OSCP is also expressed in the oocyst wall. Five days after ingestion of the blood meal, the number of oocysts decreased and they were smaller in the KO parasite compared to the WT parasite. When the existing oocyst wall structural proteins PbCap380 and PbCap93 were knocked out, the number of oocysts that had formed 15 days after the blood meal decreased [24, 25]. In the PbCap93-KO parasite, oocyst size was also smaller at 15 days after the blood meal. This suggests that OSCP is involved in oocyst growth as a wall structural protein. In the OSCP-KO parasite, the number of oocysts decreased as more days had passed since the blood meal. Accordingly, oocysts that had formed might have quickly been eliminated by the mosquito’s immune defenses during the early oocyst stage. Similar results were also observed in the PbCap380-KO parasite. In the OSCP-KO parasite, electron microscope images demonstrated thinning of the oocyst wall and delaying of sporozoite differentiation. It appears that oocyst wall structural proteins function by covering the oocyst to hide it from the mosquito’s immune defenses. Therefore, thinning of the oocyst wall causes the oocyst to be recognized by the mosquito’s immune defenses, resulting in destruction of the oocyst [25]. Moreover, thinning of the oocyst wall affects differentiation of the sporozoite, with some sporozoites being immature in the KO oocysts. This suggests that OSCP is also involved in oocyst maintenance.

Ookinete movement speed decreased after the α-1 antibody was exposed to the 926–939 amino acid part of OSCP. In addition, when mosquitoes ingested infected blood containing anti-OSCP α-1 and α-2 antibodies, the number of oocysts decreased 2 days after the blood meal, and infection rates also decreased 5 days after the blood meal. These results suggest that anti-OSCP α-1 antibodies that recognize the 926–939 amino acid block transmission. Ookinetes travel passively through the midgut lumen until they reach the midgut cell surface. By connecting to the midgut cells, the ookinete is able to exhibit motility and travel to below the midgut basal lamina [36]. This suggests that antibodies in the ingested blood attached to the matured ookinete surface and lowered ookinete-midgut affinity, thereby reducing movement speed and blocking transmission. The changes to the three-dimensional structure of OSCP due to antibody binding appeared to directly affect ookinete motility.

To achieve TB in a parasite, a control using a TB vaccine (TBV) for proteins expressed on the parasite surface in the mosquito stages has been proposed. There are two kinds of TBVs: pre-fertilization TBVs target proteins expressed on female gametocyte and female gamete surfaces, while post-fertilization TBVs target proteins that are expressed on zygote and ookinete surfaces [29, 37]. The anti-OSCP antibodies decreased the gliding speed of WT ookinetes *in vitro*. Moreover, the anti-OSCP antibodies reduced the number of oocysts 2 days after the blood meal. This implies that OSCP could be a new candidate for a post-fertilization TBV protein.

Previous results have shown that OSCP is involved in ookinete invasion into the midgut cells and in maintaining the structure of the oocyst wall, suggesting that anti-OSCP antibodies would block transmission. Only knocking out OSCP did not cause any major changes to morphology and did not completely stop movement or transmission to mosquitoes. Based on this, it can be predicted that even if OSCP is knocked out, some proteins may function to compensate for its loss. Using anti-OSCP antibodies decreased infection rates to mosquitoes by 25.4% on day 15 after the blood meal. The reason why anti-OSCP antibodies did not completely block transmission may be because of the same reason as for KO parasites. In case of inhibition of OSCP by antibodies, some proteins may function to compensate for its loss. To achieve a complete blocking efficacy, it is desirable to search for epitopes that have better effects to reduce infection rates of mosquitoes. A previous model has shown that malaria can be eradicated if transmission is blocked by 35% [38]. Therefore, better epitopes of OSCP and/or its related proteins that can block transmission from mosquitoes by 35% are required. Going forward, to analyze the mechanism for ookinete motility changes, future studies should address whether complexes had formed and whether interaction with mosquito-derived proteins is seen. Further research also needs to investigate whether anti-OSCP antibodies function similarly in blocking transmission to *P. falciparum*.

## Conclusions

In summary, we identified that the OSCP protein has functions in both the ookinete motility and oocyst growth. In addition, anti-OSCP antibodies reduce ookinete speed and have an inhibitory effect on oocyst formation. Further studies are needed to determine the potential role of the OSCP protein and whether anti-OSCP antibodies can be used as a TB tool for malaria.

## Data Availability

Data available on request from the authors.

## Abbreviations

CelTos: Cell-traversal protein for ookinetes and sporozoites
CTRP: Circumsporozoite- and TRAP-related protein
GCβ: Guanylate cyclase β
IFA: Immunofluorescence assays
KO: Knockout
LYSC1: Lysozyme c-1
MMP1: Matrix metalloprotease 1
OKM: Complete ookinete medium
OSCP: Ookinete surface and oocyst capsule protein
PbCap380: *Plasmodium berghei* Oocyst capsule protein 380
PbCap93: Oocyst capsule-associated protein 93 of *Plasmodium berghei*
PIMMS2: *Plasmodium* Invasion of mosquito midgut screen candidate 2
PPLP3-5: *Plasmodium* Perforin-like protein 3–5
PM: Peritrophic matrix
RT: Room temperature
SOAP: Secreted ookinete adhesive protein
TB: Transmission blocking
TBV: Transmission-blocking vaccine
WT: Wild type
